# KRAS-dependent suppression of MYC enhances the sensitivity of cancer cells to cytotoxic agents

**DOI:** 10.18632/oncotarget.14929

**Published:** 2017-02-01

**Authors:** Irene Ischenko, Jizu Zhi, Michael J. Hayman, Oleksi Petrenko

**Affiliations:** ^1^ Department of Molecular Genetics and Microbiology, Stony Brook University, Stony Brook, NY 11794, USA; ^2^ Department of Pathology, Stony Brook University, Stony Brook, NY 11794, USA

**Keywords:** NSCLC, KRAS, MYC, MAPK, cytotoxicity

## Abstract

KRAS is the most commonly mutated oncogene, frequently associated with some of the deadliest forms of cancer. However, the need for potent and specific KRAS inhibitors remains unmet. Here, we evaluated the effects of selected cytotoxic agents on oncogenic KRAS signaling and drug response. The data provided new insights into the functional interaction between the KRAS and MYC pathways and revealed key differences between WT and mutant KRAS expressing cells. Systematic investigation of non-small cell lung cancer cell lines revealed that KRAS mutation can paradoxically increase the sensitivity of cells to cytotoxic agents. We identify MYC as a key regulator of the cellular stress responses and tumor cell viability as MYC expression was suppressed in drug-sensitive but not resistant cells. Furthermore, this suppression was driven by hyperactive KRAS/MAPK signaling. Our findings support a direct link between MYC and cancer cell viability, and raise the possibility that inactivation of MYC may be an effective therapeutic strategy for KRAS mutant tumors across various cancer types.

## INTRODUCTION

Lung cancer is a leading cause of cancer-related death worldwide, accounting for ~1.6 million deaths annually. Lung cancers are generally divided into two main categories: small cell carcinoma and non-small cell carcinoma (NSCLC). The NSCLCs account for nearly 80% of all lung cancers and can be subdivided into adenocarcinoma (ADC; 50% of all cases), squamous cell carcinoma (SCC; 40%) and large cell carcinoma (10%). Recent advances in whole genome sequencing have further delineated NSCLCs as a group of distinct diseases with genetic and cellular heterogeneity. It is now evident that NSCLCs harbor large numbers (>200) of mutations, although as few as three driver gene mutations may be sufficient for the appearance of terminal cancer [1]. Most lung cancers either lack an identifiable oncogene, or tend to affect genes that can be clustered into a smaller number of signaling pathways and processes. Among these, mutations in KRAS (>30%), EGFR (~15%) and ALK (~5%) prevail in lung ADCs, whereas mutations affecting the PI3K pathway (~50%) prevail in lung SCCs [2, 3]. Mutations in other genes, such as ERBB2, MET, BRAF, MAP2K1 and NF1, are almost always mutually exclusive with KRAS or PIKC3A mutations; however, the frequencies of these mutations are low. To put these data in context, mutations that result in increased RAS or PI3K activity are present in ~50% of NSCLCs. From a therapeutic standpoint, such tumors are more likely to be amenable to targeted therapies than tumors with rare combinations of mutations but no identifiable oncogene. In turn, targeted therapies are expected to be more effective than chemotherapy and radiation, the mainstays of cancer treatment today. Recent discoveries have unveiled an impressive list of the RAF/MEK/ERK and PI3K/AKT pathway inhibitors, offering a new treatment paradigm for cancer patients. However, despite the initial promise, most of the responses to these pathway inhibitors have been partial and short lived.

The presence of oncogenic KRAS mutations has become widely accepted as a negative predictor for treatment outcome. Resistance and off target toxicity are major challenges in the development of clinically suitable drugs [4]. Cancer cells frequently respond to standard treatments by readjustment of signaling networks or through acquisition of bidirectional conversions between KRAS-dependent (drug-sensitive) and independent (drug-resistant) cell states [4, 5]. There is demand for novel approaches to identify mechanisms responsible for KRAS-mediated drug resistance and determine which signaling nodes are suitable for treatment, as well as which targets to select within these nodes. Arguably, the role of the proto-oncogene MYC in drug resistance is one of the biggest unanswered questions concerning KRAS-driven cancers. Current models posit that MYC is essential for KRAS-driven cancer [6]; RAS activation stabilizes MYC [7]; in turn, MYC renders cells vulnerable to DNA damage and apoptosis [8]. Clearly, these models appear to contradict one another. Perhaps the most obvious contradiction is that the degree and duration of oncogenic RAS activation would have profound effects on MYC protein accumulation and thus enhance rather than decrease tumor sensitivity to chemotherapy or radiotherapy. Also, while most early studies utilized the activated form of HRAS (generally referred to as RAS), each RAS isoform (HRAS, KRAS and NRAS) has a distinct biological function, and KRAS mutations are much more common in human cancers than NRAS and HRAS mutations.

To resolve these apparent contradictions, we evaluated the effects of selected cytotoxic agents on the inhibition of oncogenic KRAS signaling and drug response. Systematic investigation of NSCLC cell lines revealed that KRAS mutation can paradoxically increase the sensitivity of cells to cytotoxic agents. We reveal functional separation between resistance mechanisms and KRAS mutation status and demonstrate that drug-induced cytotoxicity of KRAS mutant cancer cells is contingent on MYC inhibition. MYC is suppressed in drug-sensitive cells, and this suppression is mediated by increased and prolonged activation of the MAPK/ERK pathway. Our results provide new insight into the complex nature of KRAS-MYC interactions, the majority of which do not fall into easily recognizable within-pathway relationships. This more comprehensive understanding of KRAS-MYC relationships will inevitably be informative for the goal of manipulating KRAS signal activity for therapeutic purposes.

## RESULTS

### Targeting of KRAS by combing MEK, PI3K and HDAC inhibitors overcomes drug resistance in lung cancer cells

**Figure 1:**
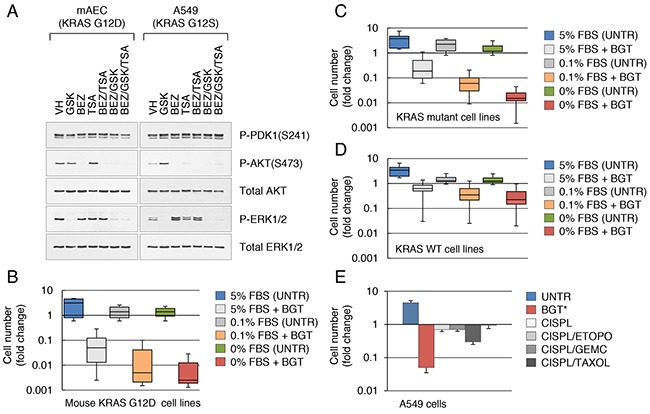
Targeting KRAS in combination with HDACs overcomes drug resistance in lung cancer cells **A**. Western blot analysis of mouse KRAS G12D lung epithelial cells and human A549 lung cancer cells treated with the indicated inhibitors at 0.1 μM for 24 hrs. **B**. Clonal KRAS G12D cell lines (n=17) were maintained in DME supplemented with different concentrations of FBS and treated with BGT inhibitors at 0.1 μM for 3 d. Fold change in cell numbers relative to input cells is shown. Error bars represent the standard deviation. P-values were <0.05 for each treatment group. **C, D**. Human KRAS mutant (n=8) (C) or KRAS/BRAF WT NSCLC cell lines (n=12) (D) were maintained in DME supplemented with different concentrations of FBS and treated with BGT inhibitors at 0.1 μM for 3 d. Fold change in cell numbers relative to input cells is shown. Error bars represent the standard deviation. P-values were <0.05 for each treatment group. **E**. A549 cells were treated for 3 d with BGT at 0.2 μM or with the indicated cisplatin-based drugs combination at 10 μM each. Fold change in cell numbers relative to input cells is shown. Error bars represent the standard deviation. *Statistically significant, p<0.05.

A large body of evidence indicates that inhibition of oncogenic KRAS by either genetic (shRNA) or pharmacological approaches delays, but does not prevent, tumor growth due to the ineffective induction of cell death [9]. The basis of this remains unclear but is thought to be influenced by the mutational complexity of tumors. To identify the particular vulnerabilities of KRAS mutant cancer cells, we have developed a panel of primary lung epithelial cells that carry a conditional mutant allele of the KRAS gene (KRAS G12D) on a p53-null genetic background. These cells can be clonally expanded in culture and produce tumors in mice, consistent with the role of KRAS in lung carcinogenesis [10]. As signaling through the RAS/MAPK and PI3K pathways is required to sustain KRAS-induced lung tumorigenesis [11], we sought to assess the efficacy of combined targeting of these pathways in our cell system. To that end, we screened our KRAS G12D cell lines for growth inhibition and induction of cell death after exposure to chemical inhibitors of MEK (PD0325901 and GSK1120212), PI3K (BEZ235 and GDC0941) and IGF1R (OSI-906 and GSK1904529A). Of these, Trametinib (GSK1120212) is FDA-approved for melanoma; BEZ235 is a dual PI3K and mTOR inhibitor, while GDC0941 is an inhibitor of class I PI3K (http://www.cancer.gov). Recent data suggest that insulin-like growth factor 1 receptor (IGF1R) exerts dominant control over PI3K signaling in human KRAS mutant cancers [12, 13]. All these MEK/PI3K inhibitor combinations exhibited marked downregulation of MAPK and PI3K signaling, as assessed by the levels of activated ERK and AKT (Figure 1A). However, there was no significant cytotoxicity against tumor cells (Supplementary Figure 1A, B). We and others have recently reported that drug-tolerant cells can be ablated via co-targeting the MAPK and PI3K pathways and histone deacetylase (HDAC) inhibition [14, 15]. We also demonstrated that targeting MEK and PI3K in combination with HDACs reduces the self-renewal of PDAC cells harboring the mutant KRAS allele (KRAS G12D) and blocks cancer metastasis *in vivo* [15]. Applying this treatment regimen to the KRAS G12D lung cancer cell lines likewise resulted in acute sensitivity to MEK/PI3K/HDAC inhibitor combination. The strongest cytotoxic effects were obtained with GSK1120212, BEZ235 and trichostatin A (TSA), a classical inhibitor of class I and II HDACs (Figure 1B). Short-term use of the BEZ/GSK/TSA drug combination (hereafter referred to as BGT) caused growth inhibition and cell death of up to 90% of KRAS mutant cancer cells (Supplementary Figure 1A, 1B). At low concentrations (below 0.2μM), these drugs were relatively non-toxic to normal lung cells (Supplementary Figure 1C). Thus, targeting of KRAS by combing MEK, PI3K inhibitors and TSA overcomes drug resistance in lung cancer cells.

### Targeting KRAS signaling pathways in human lung and colon cancer cells

We next evaluated the drug sensitivity of a panel of >20 human NSCLC cell lines representing the genetic diversity of lung cancer (Table S1). Eight of these cell lines have activating KRAS mutations (G12A, G12C, G12S, G12V or Q61H), while other cell lines contain wild-type RAS alleles (KRAS, NRAS and HRAS) and are not RAS-activated (Table S1). All of the cell lines were sensitive to MEK and PI3K inhibition, as assessed by the activation status of ERK and AKT (examples are shown in Figure 1A). Consistent with the above result, combinations of MEK and PI3K inhibitors exhibited marked cytostatic but not cytotoxic effects on all cell lines tested (Supplementary Figure 1B). The combined MEK/PI3K and HDAC inhibition greatly improved the outcomes. The highest viability reduction (~80%) was seen in KRAS mutant cells, whereas the lowest reduction (~20%) was found in KRAS WT cells (Figure 1C, 1D). To directly test whether expression of oncogenic KRAS is sufficient to confer drug resistance, cells were maintained in medium containing different concentrations of serum, ranging from 5% to 0%, and their drug responses were assessed after treating with cytotoxic compounds (Figure 1C, 1D). Tumor cell viability in serum-depleted media did not change for up to 6 days. However, we observed a further decrease of the viability of BGT-treated cells in the low range of serum concentrations, with ~98% of KRAS mutant cells succumbing to cell death after 3 days of treatment (Figure 1C). Hence, factors present in serum, rather than KRAS alone, provide protection from the cytotoxic effects of these drugs (further discussed below). It is interesting to note that KRAS WT cell lines were found to have varying levels of sensitivity and resistance to BGT treatment (Figure 1D). Whether this reflects additional mutations that can affect RAS signaling is presently unclear. Extending our analysis, we tested the impact of MEK/PI3K/HDAC inhibition on a panel of colorectal (CRC) cells carrying single and compound KRAS, BRAF and PI3K mutations (Table S1). We observed a relatively uniform response across all cell lines tested, as the BGT inhibitor combination had a measurable cytotoxic activity against KRAS/PI3K mutants and BRAF/PI3K mutants, as well as those without dual mutations (Supplementary Figure 1D). Overexpression of exogenous mutant KRAS likewise conferred enhanced drug sensitivity on NSCLC cell lines with WT RAS alleles (Supplementary Figure 2C). Direct comparison showed that low doses of BGT (0.2 μM) were more effective than considerably higher concentrations (10 μM) of the currently accepted gemcitabine and cisplatin-based combinations (Figure 1E and Supplementary Figure 2E). We therefore used this drug combination, alongside gemcitabine and cisplatin (hereafter referred to as GC), as a tool to identify the resistance mechanism(s) of lung cancer cells and reveal targetable pathways to overcome this resistance.

### Functional separation between resistance mechanisms and KRAS mutation status

**Figure 2:**
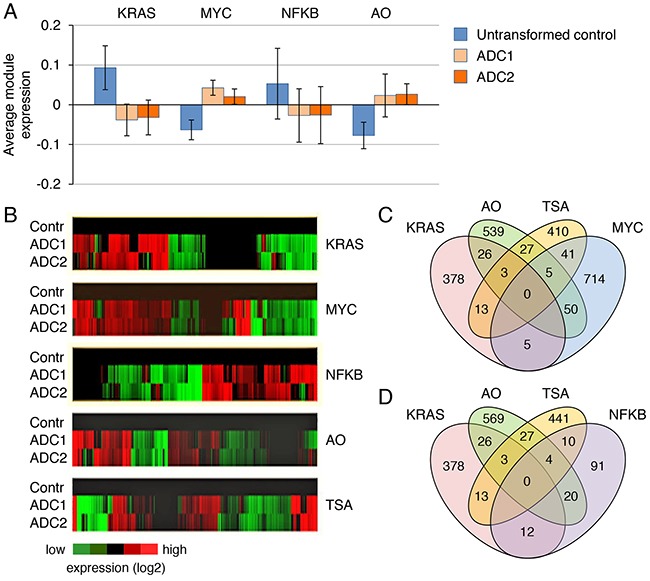
Functional separation between KRAS mutation status and resistance mechanisms **A**. Average gene expression values (log2) for each module in normal, untransformed lung epithelial cells, primary and secondary KRAS G12D mutant lung adenocarcinomas (ADC1 and ADC2, respectively). **B**. Heatmaps of differentially expressed genes for each module shown in (A). **C, D**. Venn diagrams depicting overlaps between the indicated modules. The number of genes shared between each pair of modules is shown.

To better understand how the mutation status of KRAS influences survival signaling in cancer cells, we utilized data obtained from gene expression profiling of mouse KRAS G12D-induced lung adenocarcinomas and control untransformed cells, which we previously reported [10]. The number of genes whose expression was changed more than four- or twofold in KRAS-transformed cells compared with controls was ~500 and 5000, respectively. Relying on publicly available data sets (http://www.broadinstitute.org), we defined gene expression modules for KRAS, MYC, NFkB, TSA, and the onset of apoptosis (AO) based on retrieved human cancer gene sets. Our rationale for choosing these modules as relevant to the KRAS pathway in cancer was as follows: it has been demonstrated that MYC integrates RAS and PI3K signals and that both under- and overexpression of MYC can lead to cancer cell death [16, 17]. Likewise, the genetic or pharmacological inhibition of NFkB enhances drug-induced apoptosis in lung cancer mouse models [18, 19]. Although drug-induced apoptosis is often associated with activation of JNK-AP1 signaling, the recruitment of AP1 (activator protein-1) is viewed as a consequence, rather than a cause, of drug-induced cell death [20]. We found that other candidate gene expression modules that could potentially influence the cell death pathways, such as the E2F, SRF (serum response factor), STAT3 and TGFB/SMAD, were not consistently activated by KRAS G12D in lung ADC (Supplementary Figure 3). Using these module definitions, we then calculated mean module activities for the control and tumor-derived KRAS G12D cells (Figure 2A, 2B). As shown in Figure 2B, the KRAS and MYC signatures are similar in ADC1 and ADC2 as opposed to untransformed controls. However, although the KRAS module is made up of >400 genes that are either upregulated (44%) or downregulated (56%) in the tumor cells, while the MYC module is comprised of >800 genes that are upregulated (54%) or downregulated (46%), we found that for each module, the up- and downregulated genes are different. Only a small proportion (<2%) of these genes overlap (Figure 2C). Earlier pathway-based analyses of primary lung cancers and NCI60 cell lines also did not identify direct interactions involving RAS and MYC [21, 22]. Likewise, the genes that were up- or downregulated in the NFkB module were distinct from those regulated by either KRAS or MYC (Figure 2D). Thus, the genes belonging to each of the three modules (KRAS, MYC and NFkB) in KRAS-induced ADCs are regulated separately and control different aspects of the malignant phenotype. While surprisingly small overlap was observed between the KRAS/AO and NFkB/AO modules, we found a modest but statistically significant overlap between the MYC/AO and MYC/TSA modules (55 and 46 genes, respectively; p values of <0.0001 for each), suggesting a possible component of drug-induced cell death (Figure 2C). The obtained prediction of antipodal effects of KRAS and MYC induction on the drug sensitivity of cancer cells was then tested experimentally.

### Constitutive activation of KRAS does not sustain high levels of MYC

**Figure 3:**
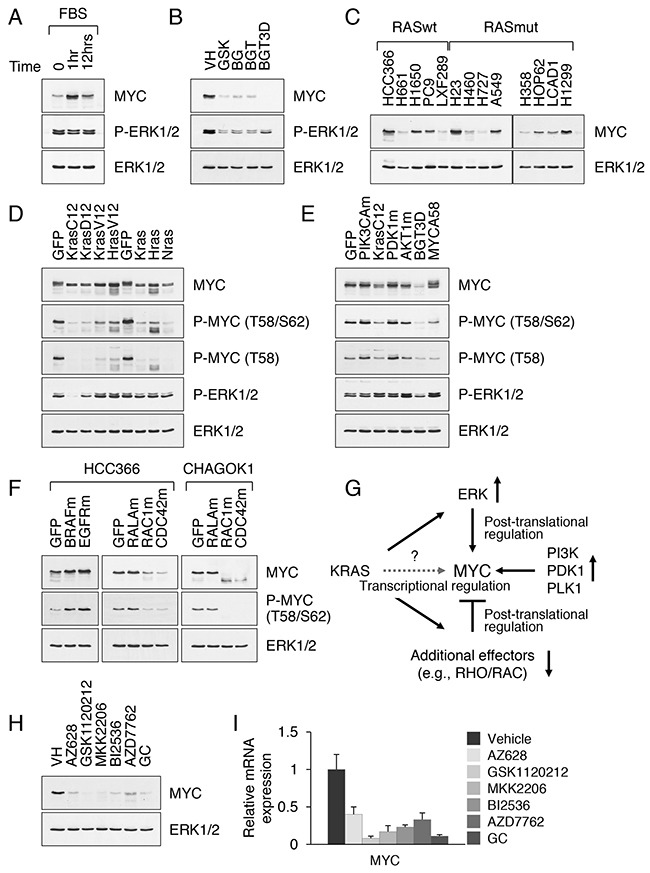
Constitutive activation of KRAS does not sustain high levels of MYC **A**. Western blot analysis of nuclear extracts from A549 cells cultured under serum-free conditions for 24 h and then stimulated with serum for the indicated time periods. **B**. MYC expression in nuclear extracts from A549 cells treated with the indicated inhibitors at 0.1 μM for 3d. For controls (BGT3D), cells treated with BGT and then released into drug-free medium for 3d are shown. **C**. MYC expression in nuclear extracts from KRAS WT and mutant NSCLC cell lines. **D**. MYC expression in nuclear extracts from CHAGO-K1 cells stably transduced with the activated or wild-type RAS isoforms. **E**. MYC expression in nuclear extracts from A549 cells stably transduced with GFP, PIK3CA H1047R (PIK3CAm), myrPDK1 (PDK1m), myrAKT (AKTm) and MYC T58A. **F**. MYC expression in nuclear extracts from HCC366 and CHAGO-K1 cells stably transduced with GFP, BRAF V600E (BRAFm), EGFRvIII (EGFRm), RAC1 G12V (RAC1m) and CDC42 Q61L (CDC42m). **G**. A proposed mechanism of MYC regulation by oncogenic KRAS. **H, I**. MYC protein (H) and mRNA expression (I) in A549 cells treated with the indicated inhibitors for 3 days. Relative levels of MYC mRNA were normalized to the expression HPRT mRNA.

MYC is an early response gene, not expressed in growth arrested cells but rapidly induced in response to growth factor stimulation [23]. Tissue-specific knockout studies revealed that MYC functions in cell proliferation, metabolism and maintenance of self-renewal in several types of stem cells [9]. Suppression of MYC in cancer cell lines reduces cell viability [24]. The current model posits that RAS signaling affects MYC by two basic mechanisms: PI3K inhibits phosphorylation of MYC at T58, which blocks its proteolysis by the ubiquitin proteasome system, while activated ERK phosphorylates MYC at S62, which increases its stability [25, 7]. We confirmed that growth factor-induced signaling mediates the induction of MYC expression in cells bearing constitutively active KRAS (Figure 3A). We also confirmed that the potent inhibitors of MEK and PI3K block tumor cell growth and MYC protein expression (Figure 3B). Somewhat surprisingly, we found that the steady state levels of MYC vary in NSCLC cell lines regardless of the presence or absence of oncogenic KRAS mutations, ranging from the levels seen in normal cells (e.g. NIH3T3 and IMR90 cells, around or less than 5,000 molecules per cell) to the levels seen in many tumor cells (e.g. HeLa cells, >30,000 molecules per cell) [26, 27]. For instance, high levels of MYC were detected in HCC366 and H23 cell lines, which have previously been reported to contain amplified *myc* locus (Figure 3C). Other NSCLC cells, as well as KRAS mutant CRC and PDAC cells, frequently showed a lower, physiological level of MYC expression (Figure 3C and Supplementary Figure 4A). Phosphorylation of MYC at T58 and S62 was consistently present in all tumor cells (Supplementary Figure 4B). We therefore sought to examine whether MYC expression correlates with RAS activation status in primary and cancer cell lines.

To that end, we analyzed primary LSL KRAS G12D p53KO (inactive KRAS G12D allele) and KRAS G12D p53KO (active KRAS G12D allele) lung epithelial cells or tumors derived from these cells. We found that MYC levels remained unchanged regardless of whether cells expressed the KRAS G12D oncogene (Supplementary Figure 4C). We generated human NSCLC cell lines stably transduced with either activated or wild-type RAS isoforms (HRAS, KRAS or NRAS) (Figure 3D). We compared the three most common KRAS mutations in lung ADC, namely G12C (42% of total KRAS mutations), G12V (20%) and G12D (17%). Each of these mutations generates a distinct signaling output, as KRAS G12C signals primarily through RAL and KRAS G12D signals primarily through PI3K as opposed to other downstream effectors [28]. The analysis revealed that two pools of MYC exist in KRAS-transformed cells (the unstable, T58-phosphorylated pool, and the more stable, non-phosphorylated pool), in agreement with previous reports [29]. The expression of activated RAS mutants, in the background of wild-type RAS alleles, caused a decrease in phospho-T58 MYC without having a major stabilizing effect on basal levels of MYC, possibly with the exception of HRAS G12V (Figure 3D). Moreover, ectopic expression of KRAS G12C, as opposed to KRAS G12D and KRAS G12V mutants, induced reduction of MYC levels in ~50% of all cell lines examined (Figure 3E). Ectopic expression of WT RAS isoforms also caused a decrease in phospho-T58 MYC, implying that overactive RAS signaling can either suppress the phosphorylation of MYC at T58 or enhance the degradation process (Figure 3D). These data imply that the constitutive activation of KRAS alone does not sustain high levels of MYC. Supporting this notion, endogenous mutant KRAS was inefficient in inducing MYC expression in A549 cells maintained in media without serum (Figure 3A) or growth arrested following treatment with MEK/PI3K inhibitors (Figure 3E). Furthermore, the MYC T58A mutant that cannot be phosphorylated on T58 was only moderately more stable than endogenous MYC in KRAS mutant A549 cells (Figure 3E). That is, although MYC T58A is more stable than WT MYC, it does not accumulate appreciably in the context of mutant KRAS.

On the other hand, expression of constitutively active PIK3CA H1047R and Myr-PDK1 mutants stabilized MYC by ~2-fold, apparently by blocking its phosphorylation-dependent degradation rather than phosphorylation on the T58 or S62 sites per se, since there was an increase in overall MYC levels, as well as in the unstable, phosphorylated form of MYC (Figure 3E). Likewise, stable expression of BRAF V600E confirmed a direct link between the V600E mutation (and hence the activation of MAPK/ERK pathway) and MYC stabilization (Figure 3F). Expression of the cancer-derived EGFR mutant, commonly known as EGFRvIII, also caused an increase in MYC above background levels (Figure 3F). Of note, both BRAF V600E and EGFRvIII promoted the stability of MYC either singly phosphorylated at T58 or doubly phosphorylated at T58 and S62 (Figure 3F). In contrast, expression of activated RALA, the third best characterized effector of RAS signaling in cancer, had no effect on MYC accumulation and phosphorylation (Figure 3F). The implication of these findings is that KRAS signaling controls the basal levels, but not the induced levels, of MYC (shown schematically in Figure 3G). Although growth factor receptor systems, such as those for EGF, are known to signal upstream of RAS, they nonetheless account for differential expression of MYC in KRAS-transformed cells. This may explain why the development of KRAS-driven cancers is strongly aided by elements of the tumor microenvironment, including growth factors and cytokines [30, 31, 32]. Moreover, wild-type, but not oncogenic, RAS regulates signaling from upstream RTKs [33].

We therefore sought to determine which of the known KRAS effector pathways may act to limit the accumulation of MYC protein. Logical candidates include RHO family members RAC1 and CDC42, which control pathways downstream of RAS [34, 35]. Both RAC1 and CDC42 exert their effects in part by negatively regulating MYC through PAK-mediated phosphorylation and degradation [36, 37]. RHO signaling has also been implicated in the regulation of GSK3-mediated MYC T58/S62 degradation pathway [38]. The expression of active RAC1 G12V and CDC42 Q61L indeed caused a profound and sustained decrease in the levels of MYC, even in the presence of FBS (Figure 3F). The addition of the proteasome inhibitor MG132 restored MYC levels (Supplementary Figure 4D), confirming that MYC is regulated at posttranslational levels through RAC1/CDC42 and their effector pathways. In addition, serum-induced expression of MYC was severely impaired, at both the transcriptional and posttranscriptional levels, by pharmacological inhibition of RAF (with AZ628), MEK (GSK1120212), AKT (MKK2206), PLK1 (BI2536) and CHK1 (AZD7762) (Figures 3H and 3I), highlighting the redundancy of signaling mechanisms that control MYC expression. The cytotoxic activity of gemcitabine alone or in combination with cisplatin (GC) or GSK1120212 (GEMC/GSK) also caused a sustained downregulation of MYC expression regardless of the presence of mutant KRAS (Figure 3H and Supplementary Figure 4E).

### Suppression of MYC enhances the sensitivity of cancer cells to cytotoxic agents

**Figure 4:**
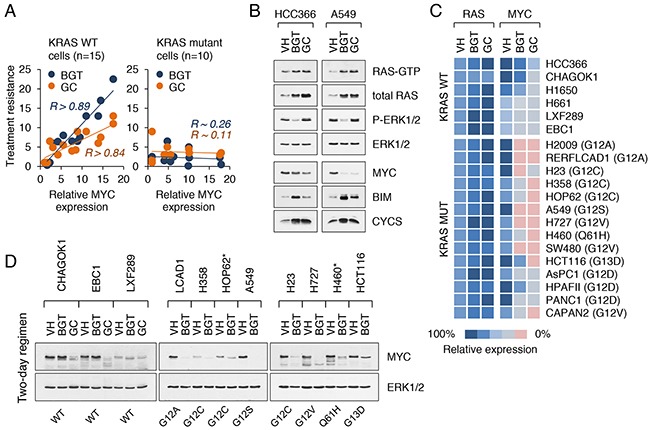
Suppression of MYC enhances the sensitivity of cancer cells to cytotoxic agents **A**. Correlation between the levels of MYC expression and drug resistance (BGT and GC treatment regimens) in KRAS WT (left) and KRAS mutant (right) cancer cell lines. Drug sensitivity was measured by the ratio of the number of live cells in the treated samples to the number of live cells in the untreated controls. *R* refers to the correlation coefficient. **B**. Western blot analysis of KRAS WT (HCC366) and KRAS mutant (A549) cell lines treated with vehicle alone, BGT inhibitors at 0.1 μM or GC at 5 μM for 2 d. RAS-GTP levels are shown. **C**. Schematic heat map showing RAS and MYC expression values in KRAS WT and KRAS mutant NSCLC, CRC and PDAC cell lines treated as in (B). Color key for expression levels is shown. Cells were maintained in DME supplemented with 5% FBS. **D**. MYC expression in nuclear extracts from NSCLC and CRC cell lines treated as in (B). Asterisks indicate BGT-resistant lines.

The observed variability in MYC expression levels among tumor cell lines with KRAS mutations prompted us to investigate whether MYC plays a role in anticancer drug resistance. To that end, we compared the effects of gemcitabine plus cisplatin (GC regimen) and BEZ/GSK/TSA treatment (BGT regimen) on NSCLC and CRC cell lines carrying wild-type or mutant KRAS alleles. We reasoned that the use of two different drug combinations can more readily identify key drug resistance pathways than each regimen separately. Among cell lines with WT KRAS (such as those in Figure 3C), there was a strong positive correlation between the expression of MYC and drug resistance (Figure 4A). In contrast, no such correlation was found for KRAS mutant cell lines (Figure 4A). At face value, these data suggested that MYC is not essential for treatment response in KRAS mutant tumor cells. However, closer examination revealed that MYC was suppressed in drug-treated cells and this suppression was more pronounced in cells harboring KRAS mutations (Figure 4B, 4C). We observed that the levels of GTP-bound (active) RAS as well as total RAS were consistently increased in cells exposed to either BGT or GC treatment, while MYC levels declined (Figure 4B, 4C). The suppression of MYC by these drugs appeared to be specific, since expression of other early response genes, such as FOS, FOSB, JUN, JUND, and MYCN, was either not affected or increased (Supplementary Figure 5A). Overall, drug sensitive cell lines (such as A549 or H727) could be distinguished from the resistant cell lines (such as HOP62 or KRAS/PIK3CA double mutants H460) based on a greater degree of MYC suppression (Figure 4C, 4D).

### Drug-induced cytotoxicity of KRAS mutant cancer cells is contingent on MYC inhibition

**Figure 5:**
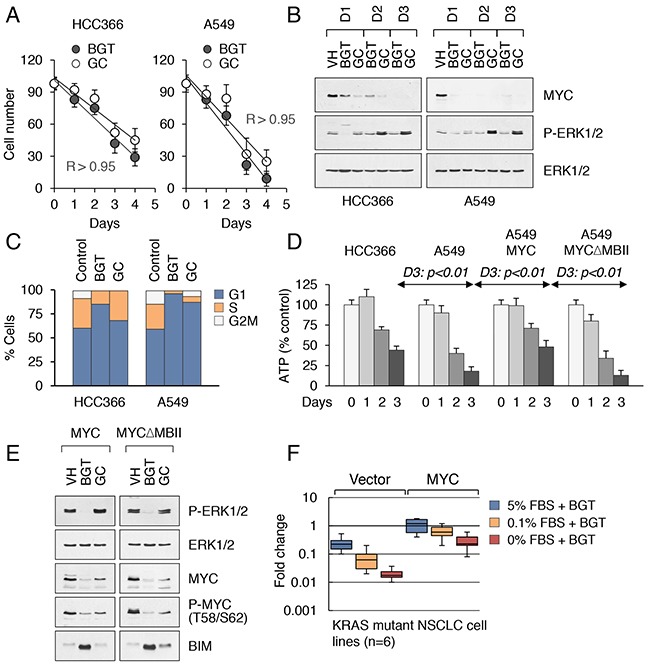
Drug-induced cytotoxicity of KRAS mutant cancer cells is contingent on MYC inhibition **A**. Correlation between treatment duration and efficacy (BGT and GC treatment regimens) in HCC366 (left) and A549 (right) cell lines. Pearson correlation coefficients for each drug treatment condition (R > 0.95). **B**. MYC expression in nuclear extracts from HCC366 (left) and A549 (right) cell lines treated for 3 days with the indicated inhibitors. **C**. Cell cycle distribution in KRAS WT (HCC366) and KRAS mutant (A549) cell lines treated for 3 days with the indicated inhibitors. **D**. Comparison of intracellular ATP contents in HCC366, A549 cells (right panels), and A549 cells transduced with wild-type MYC or the MYCΔMBII mutant (right panels) and treated with GC at 5 μM for 3 d. Cells were maintained in DME supplemented with 0.1% FBS. **E**. Western blot analysis of A549 cells with stable expression of wild-type MYC or MYC ΔMBII mutant treated as in (B). **F**. Human KRAS mutant NSCLC cell lines (n=6) were transduced with vector alone or MYC-expressing retroviruses. Cells were maintained in DME supplemented with different concentrations of FBS and treated with BGT inhibitors at 0.1 μM for 3 d. Fold change in cell numbers relative to input cells is shown.

The inhibition of MYC expression in both treatment conditions occurred prior to the induction of cell death (Figure 5A, 5B). Both drug-sensitive and drug-resistant cell lines arrested in G1 phase (BGT regimen) and G1/S phase (GC regimen) of the cell cycle (examples are shown in Figure 5C). However, cell cycle arrest did not confer any protective effect on cell survival, as MYC suppression with either concurrent ERK inhibition (BGT regimen) or ERK activation (GC regimen) induced an increase in the levels of pro-apoptotic BIM and the release of cytochrome c (CYCS) from mitochondria (Figure 4B). The ATP content (a marker of mitochondrial dysfunction and energy crisis) in drug-treated cells fell accordingly (Figure 5D and Supplementary Figure 5B). Moreover, while mutant versus wild-type KRAS had no effect on ATP production in untreated cells, and ATP levels in both cell types dropped, they did so more precipitously and significantly in KRAS mutant than KRAS wild-type cell lines (Figure 5D and Supplementary Figure 5B). Because MYC induces genes involved in mitochondrial energy metabolism, while its depletion can aggravate the energy collapse [39], we evaluated the growth and survival of KRAS mutant NSCLC and CRC cell lines transduced with wild-type MYC or the MYCΔMBII mutant. This mutant lacks the MBII domain of MYC, which is required for transcriptional regulation by MYC family proteins [40]. Ectopic expression of either wild-type or mutant MYC did not affect the growth rate in any of the cell lines (data not shown). However, expression of wild-type but not mutant MYC attenuated drug-induced effects, such as ATP depletion and induction of BIM upon GC treatment (~2 fold, Figure 5D, 5E). Furthermore, while expression of WT MYC conferred resistance on drug-treated cells (Figure 5F), expression of MYCΔMBII or depletion of MYC to approximately 30-50% using retrovirus-delivered shRNAs reduced the resistance of cells (Supplementary Figure 5C). It is noteworthy that drug treatment reduced LTR-driven MYC expression to a lesser extent compared with the suppression of endogenous MYC (Supplementary Figure 6A). However, enforced MYC expression did not induce apoptosis in epithelial cancer cells upon growth factor withdrawal (Supplementary Figure 6A). Treatment of serum-starved cells was accompanied by a proportional reduction in the levels of both endogenous and ectopic MYC proteins and subsequent growth arrest, thus preventing the cell from entering mitosis (Supplementary Figure 6B, 6C). Therefore, the induction of cell death is not a stress response of the cells due to *myc* deregulation. This may reflect the fact that the majority of human lung and colon cancer cell lines (>80%) do not contain WT TP53 alleles (Supplementary Table 1).

### Activation of ERK facilitates MYC suppression under drug induced stress conditions

**Figure 6:**
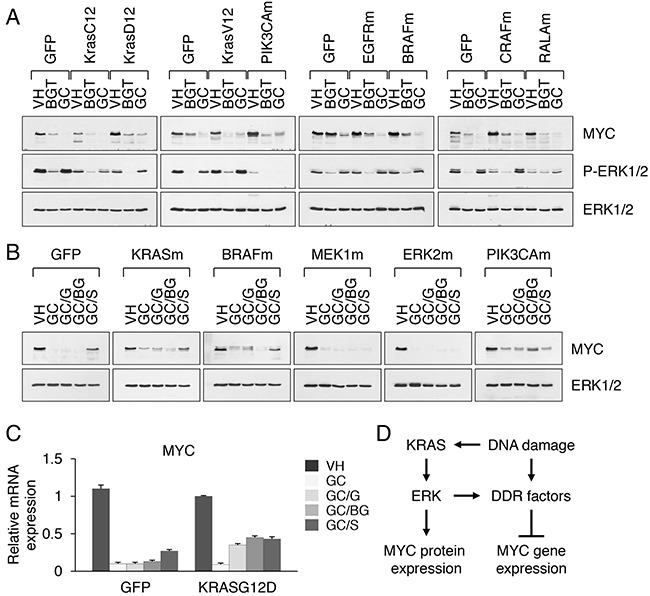
Activation of ERK facilitates MYC suppression under drug induced stress conditions **A**. MYC expression in nuclear extracts from HCC366 cells transduced with GFP, mutant KRAS (G12D, G12C or G12V), PIK3CA H1047R (PIK3CAm), EGFRvIII (EGFRm), BRAF V600E (BRAFm), CRAF 22W (CRAFm) or RALA Q75L (RALAm). Cells were treated with BGT inhibitors at 0.1 μM or GC at 5 μM for 2 d. **B**. MYC expression in nuclear extracts from HCC366 cells transduced with GFP, KRAS G12D (KRASm), BRAF V600E (BRAFm), MEK1 C121S (MEK1m), ERK2 R67S D321N (ERK2m) or PIK3CA H1047R. Cells were treated with vehicle alone or GC at 5 μM for 2 d in the presence or absence of GSK1120212 (GC/G), BEZ235 plus GSK1120212 (GC/BG) and SCH772984 (GC/S) at 0.1 μM each. **C**. MYC mRNA expression in HCC366 cells transduced with vector alone or KRAS G12D-expressing retroviruses and treated with the indicated inhibitors for 2d. Relative levels of MYC mRNA were normalized to the expression HPRT mRNA. **D**. A proposed mechanism of MYC regulation by ERK and DNA damage response (DDR) signaling.

To explore systematically the apparent antagonism between KRAS and MYC pathways and understand how cytotoxic drugs alter MYC expression and cancer cell viability, we measured their effects in isogenic NSCLC cell lines with stable expression of exogenous KRAS mutants (G12C, G12D and G12V) as well as upstream (EGFR) and downstream (PIK3CA, BRAF, CRAF, RALA) components of the RAS pathway (Figure 6A). The analysis revealed the following themes: 1) compared with GC regimen, BGT treatment was less effective at suppressing MYC expression, despite its potent inhibition of ERK activation; 2) the extent of MYC suppression in cell lines expressing the KRAS G12C or KRAS G12V mutants was stronger than that in cells with the KRAS G12D mutant; 3) in contrast, the constitutively active mutant PIK3CA H1047R dramatically reduced phosphorylation of ERK, but no detrimental effect on MYC expression was detected (Figure 6A). Likewise, mutations in EGFR, BRAF or CRAF did not alter the extent of change in MYC (Figure 6A). Due to the overall small number of NSCLC and CRC cell lines with G12D mutation, we explored a panel of PDAC cell lines (AsPC1, HPAFII, PANC1) carrying the KRAS G12D alleles (Table S1). Among the various KRAS mutations, the G12D mutant has been reported to be a stronger inducer of PI3K than RAF/MEK/ERK [28]. Pancreatic cell lines were indeed less sensitive to BGT and GC treatment than cell lines carrying the KRAS G12C or G12V mutation, as they exhibited only a modest (30%-50%) reduction in MYC expression (Figure 4C). Together, these data imply that either the activation status of MAPK/ERK pathway has no bearing on MYC expression under drug induced stress conditions, or that excess levels of activated ERK facilitate this suppression.

To discriminate between these possibilities, we examined NSCLC cells treated with gemcitabine/cisplatin in combination with MEK inhibitor GSK1120212 (GC/G regimen) and ERK inhibitor SCH772984 (GC/S regimen). Under these conditions, blocking ERK activity indeed caused an increase in MYC mRNA and protein levels (Figure 6B, 6C). To confirm these unexpected results, we examined the sensitivity of NSCLC cells toward GC/G and GC/S treatment in the context of gain of function mutations in the MAPK/ERK pathway. Notably, NSCLC cells carrying KRAS G12D or BRAF V600E mutants were more sensitive to MEK inhibition than control GFP-transduced cells, as evidenced by partly restored MYC expression upon GC/G treatment compared to GC alone (Figure 6B, C). In contrast, NSCLC cells carrying activating mutations in MEK1 (C121S) or ERK2 (R67S D321N) failed to restore MYC expression (Figure 6B). Prior studies have shown that MEK1 C121S or functionally similar mutations confer resistance to MEK inhibition *in vitro* [41, 42]. A distinctive feature of sustained MAPK/ERK signaling is thus the dual role in MYC regulation under normal and stress conditions. In cells with DNA damage, hyperactivation of ERK blocks MYC expression and prevents cell division. This can be achieved through several mechanisms, including the phosphorylation and activation of DNA damage response (DDR) factors, decreased gene transcription and modulation of post-transcriptional processes. Thus, in addition to their various functions during transcriptional activation, ERK kinases are known to interact with the RNA polymerase II C-terminal domain (RNAPII CTD) and to phosphorylate S5 in RNAPII CTD, thus establishing a stalled form of RNAPII [43, 44]. Conversely, inhibition of ERK activation partly relieves this block (shown schematically in Figure 6D). Consistent with this hypothesis, we found that combining GC treatment with MEK/ERK inhibition reduces cell death and increases the resistance of cells to chemotherapy *in vitro* (Supplementary Figure 6D). This increase, moreover, appears to be dependent on re-expression of MYC. It is noteworthy that isogenic cell lines carrying PIK3CA H1047R mutation did not respond to MEK/ERK inhibition, as in these cells MYC levels remained largely unchanged (Figure 6B). Thus, PIK3CA controls MYC expression in a MAPK/ERK-independent or possibly parallel manner under both normal and stress conditions. This may explain marginal effects of the combination treatment using PI3K inhibitors (BEZ235 and GDC0941), PDK1 inhibitor (OSU03012) and AKT inhibitor (MKK2206) on KRAS mutant cells in comparison with GC treatment alone (Supplementary Figure 6). These results affirm the importance of MYC dependency in the context of mutant KRAS and suggest novel mechanisms of resistance to anticancer agents that may have important clinical implications.

## DISCUSSION

Until recently, there has been a general consensus that MYC plays a key role in the development of many human cancers, even though enhanced cell growth caused by MYC is countered by higher rates of apoptosis [9]. Whether MYC is also required for tumor maintenance and whether tumor cells become addicted to MYC was previously unclear. Recent studies have demonstrated that blocking MYC function may be sufficient to stop tumor growth and induce tumor regression in the well-characterized LSL KRAS G12D mouse models of NSCLC and PDAC [45, 46, 47]. Inhibition of MYC overcomes drug resistance in BRAF-driven melanoma and other human cancers [48, 49, 50]. These studies have endorsed MYC as a compelling therapeutic target. Given the challenge of inhibiting KRAS directly and the role of MYC in various aspects of cancer progression, therapy for treating KRAS-driven cancers may also be expected to depend on MYC. Our data support the notion that KRAS mutant cancer cells depend on the continued expression of MYC and drug-induced cytotoxicity of KRAS mutant cells is contingent on MYC inhibition. We sought to assess the role of oncogenic KRAS in the regulation of MYC expression in cell lines derived from lung and colorectal tumors and the effects of MYC on cellular cytotoxicity and drug sensitivity according to KRAS mutation status. To that end, we performed a large scale analysis of NSCLC and CRC cell lines carrying single and compound mutations in the KRAS, BRAF and PI3K genes. We evaluated the cytotoxic effects of the currently accepted therapeutic agents (gemcitabine plus cisplatin) and novel targeted compounds (MEK, PI3K and HDAC inhibitors) on the drug sensitivity versus resistance of KRAS mutant and KRAS wild-type cancer cells. There are several important findings in our results. First, systematic investigation of NSCLC and CRC cell lines revealed that KRAS mutation paradoxically enhances the sensitivity of cells to cytotoxic agents. We identify MYC as a key component of this process and show that MYC plays an essential cell-intrinsic role in maintaining the survival of KRAS mutant cancer cells. Second, we demonstrate that constitutive activation of KRAS does not sustain high levels of MYC. Moreover, MYC is strongly suppressed in drug-sensitive cells and this suppression is facilitated by the presence of oncogenic KRAS mutations. Third, we find that activation of ERK potentiates the cytotoxicity of gemcitabine and cisplatin in NSCLC cell lines by suppressing MYC expression. Conversely, MEK/ERK inhibition reduces the effectiveness of gemcitabine and cisplatin treatment and increases the resistance of cells to chemotherapy *in vitro*. Our findings support the idea that treatment of KRAS-driven NSCLC and CRC, and potentially the other mutant KRAS driven cancers, may benefit from the concurrent inhibition of KRAS signaling and MYC. In sum, although MYC has traditionally been regarded to be a pro-apoptotic protein, drug-induced cytotoxicity of KRAS mutant cancer cells appears to depend on MYC inhibition.

MYC expression is regulated at multiple levels, including transcription, translation and protein stability [6]. Despite clear evidence that MYC deregulation contributes to cancer, the overall MYC expression in several common malignancies, such as lung, colorectal and pancreatic cancer, remains within the normal range [3, 51, 52]. For instance, MYC genes (MYC, MYCN and MYCL) are amplified and/or overexpressed in 15–30% of small cell lung cancer (SCLC) but less frequently in NSCLC [53]. Recent data show that KRAS mutation alone does not cause activation of the MYC gene at the transcriptional level [10, 54]. This raises questions about how KRAS and other signaling proteins can stabilize MYC expression through a post-transcriptional mechanism. The prevailing model holds that the activated form of HRAS (historically referred to as RAS) enhances the accumulation of MYC activity by stabilizing the MYC protein. According to this model, PI3K inhibits phosphorylation of MYC at T58, which blocks its ubiquitin-mediated proteolysis, while ERK phosphorylates MYC at S62, which increases its stability [25, 7]. It follows that the oncogenicity of HRAS (typically referred to as RAS V12) will further augment MYC levels and activity, and therefore the signaling pathways and mechanisms used by these genes are hard to separate. In the context of MYC-driven cell cycle progression, this seems to fit with the observations that the expression of MYC in mid-G1 phase is associated with sustained RAS activity[55]. In addition, MEFs devoid of all three RAS isoforms (RAS-less MEFs) display cell cycle arrest and the repression of a series of cell cycle-related genes, including MYC [56]. However, while this model may account for the differences in MYC protein expression in normal cells, our data demonstrate that the interactions between oncogenic KRAS and MYC do not fall into easily recognizable within-pathway relationships that are commonly associated with oncogenic HRAS. Although MYC is clearly required for the maintenance of KRAS-driven cancer, our data imply that the KRAS oncogene is inefficient in sustaining expression of MYC in the absence of growth factors or in response to anticancer drug treatment. We therefore infer that KRAS and MYC are regulated separately and control different aspects of the malignant phenotype. Moreover, with MYC expression data obtained before rather than during treatment, KRAS WT but not KRAS mutant cancer cells can be classified in terms of whether they would be sensitive or resistant to cytotoxic drugs. Among RAS genes, KRAS is the most frequently mutated gene in cancer (85% of RAS-driven cancers), while HRAS is the least frequently mutated gene (3%) (COSMIC). Why KRAS mutations prevail in colorectal, lung and pancreatic cancer, while NRAS or BRAF are mutated more frequently in skin melanoma, and HRAS mutations are predominant in head and neck squamous cell carcinoma is not yet clear. Likewise, it is unclear why oncogenic mutations of PI3K are common in a broader variety of human tumors. Further investigation of these cancer driver genes will be required to reveal novel insights into MYC biology and answer the question whether MYC or MYC's target genes can be targeted for cancer therapy.

## MATERIALS AND METHODS

### Mammalian cells and reagents

We used previously described pretumor and tumor-derived KRAS G12D p53KO lung epithelial cell lines [10]. These cells were grown on gelatinized plates in CnT-17 medium (CellnTec). Human cell lines were obtained from the ATCC or from individual scientists and were not further authenticated. Cell lines were cultured in RPMI or DMEM media supplemented with 5% FBS and 1x antibiotic/antimycotic, as recommended by ATCC, unless otherwise specified. For long-term cell proliferation assays, cells were seeded into 6-well plates (4×10^5^ cells per well) and cultured both in the absence and presence of serum and drugs as indicated below. Inhibitors targeting MEK (PD0325901 and GSK1120212), PI3K (BEZ235 and GDC0941), HDAC (SAHA and TSA) (all from Selleckchem.com) were prepared as 100 μM stocks in DMSO. Cells were treated with various concentrations of the compounds for 3 days, followed by a 1 day drug-free recovery period, and their proliferation was determined by Coulter counter. For intermittent inhibition, cells were subjected to three rounds of 3 day treatment, each followed by a 3 day drug-free period, over the course of 18 days. Cell viability was measured using propidium iodide (PI) staining. ATP content was measured using CellTiter-Glo kit (Promega). Retroviral vectors encoding HRAS, KRAS, NRAS, KRAS G12C, KRAS G12D, KRAS G12V, BRAF V600E, CDC42 Q61L, CRAF 22W, EGFRvIII, ERK2 R67S D321N, MEK1 C121S, MEK2 KW71, MEK2 K101A, MYCΔMBII, MYC T58A, shMYC, PIK3CA H1047R, myrAKT, myrPDK1, myrSGK1, RAC1 G12V and RALA Q75L were purchased from Addgene. Additional retroviral vectors were described previously [10].

### Expression analysis

Western blotting was performed using antibodies against MYC (N-262, Santa Cruz), MYC-S62 (11311, SAB), MYC-T58 (11034, SAB), MYC-T58/S62 (04-217, Millipore), RAS (610001, BD); KRAS (F234, Santa Cruz), PIK3CA (C73F8), PDK1 (3062), P-PDK1 (S241), AKT (9272), P-AKT (T308), P-AKT (S473), P-ERK1/2 (4370), CRAF (9422), P-CRAF (9427), BRAF (9433), P-BRAF (2696), CHK1 (2345) (all from Cell Signaling). Whole cell extracts were prepared by lysing cells in buffer containing 10 mM TrisHCl, pH7.4, 150 mM NaCl, 1 mM EDTA, 10% glycerol, 1% Triton X100, 40 mM NaVO4, 0.1% SDS, and 1x protease inhibitors (Roche). Nuclear extracts were prepared using NE-PER nuclear and cytoplasmic extraction reagents (Thermo Scientific). Western blots were imaged and quantified using Image Studio software (LI-COR). For cell cycle analysis, cells were lifted with Trypsin, fixed in 70% ethanol, stained with PI and analyzed using FACSCalibur (BD) with CellQuest and ModFit LT software. For RNA isolation, cells were harvested with TRIzol reagent (Invitrogen). First strand cDNA synthesis was performed using SuperScript II reverse transcriptase (Invitrogen) followed by SYBR green quantitative polymerase chain reaction (qPCR). The following primer pairs were used: myc1: 5-gaaaaggcccccaaggtagttatc-3; 5-tcgtttccgcaacaagtcctcttc-3; myc2: 5-cttctctccgtcctcggattct-3; 5-gaaggtgatccagactctgac ctt-3; erk2, 5-caacccacacaagaggattgaa-3; 5-gtcgaacttgaatgg tgcttcg-3; hprt: 5-gctataaattctttgctgacctgctg-3; 5-attactttta tgtcccctgttgactg-3. Relative levels of MYC mRNA were normalized to the expression HPRT mRNA. Statistical analyses were performed using Student's t test. P ≤ 0.05 was considered statistically significant.

### Microarray analysis

We used publicly available data sets (http://www.broadinstitute.org), (http://www.bu.edu/nf-kb/gene-resources/target-genes/) and (http://amigo2.berkeleybop.org/amigo/) to retrieve gene sets comprising the KRAS, MYC, NFkB, TSA, and regulation of apoptosis modules. The BioMart (http://central.biomart.org) and the Complete List of Human and Mouse Homologs (http://www.informatics.jax.org/orthology.shtml) were used to standardize genetic nomenclature. The heat maps were generated by calculating ratios of expression in each sample vs. control. The log2 values were then supplied to the heat map function of the R statistical package. The modified Gene Set Enrichment Algorithm (GSEA) was used to perform the pathway-based analyses. Module expression analysis was conducted as described [57]. Average gene expression values (log2) of all genes were set as baseline 0. The gene expression values (log2) of each module relative to the overall average were represented as mean ± SEM. The hypergeometric test was used to calculate the probability for overlapping genes between different gene sets. The Venn diagrams were generated as described [58].

## SUPPLEMENTARY MATERIALS FIGURES AND TABLES




