# Supplementary material for: KRAS-dependent suppression of MYC enhances the sensitivity of cancer cells to cytotoxic agents
Source: Oncotarget. 2017 Feb 1;8(11):17995–8009. doi: 10.18632/oncotarget.14929 (PMC5392302; doi:10.18632/oncotarget.14929)
Supplement: Supplementary file 1 [file oncotarget-08-17995-s001.pdf]

KRAS-dependent suppression of MYC enhances the sensitivity of cancer cells to cytotoxic agents

SUPPLEMENTARY FIGURES AND TABLE

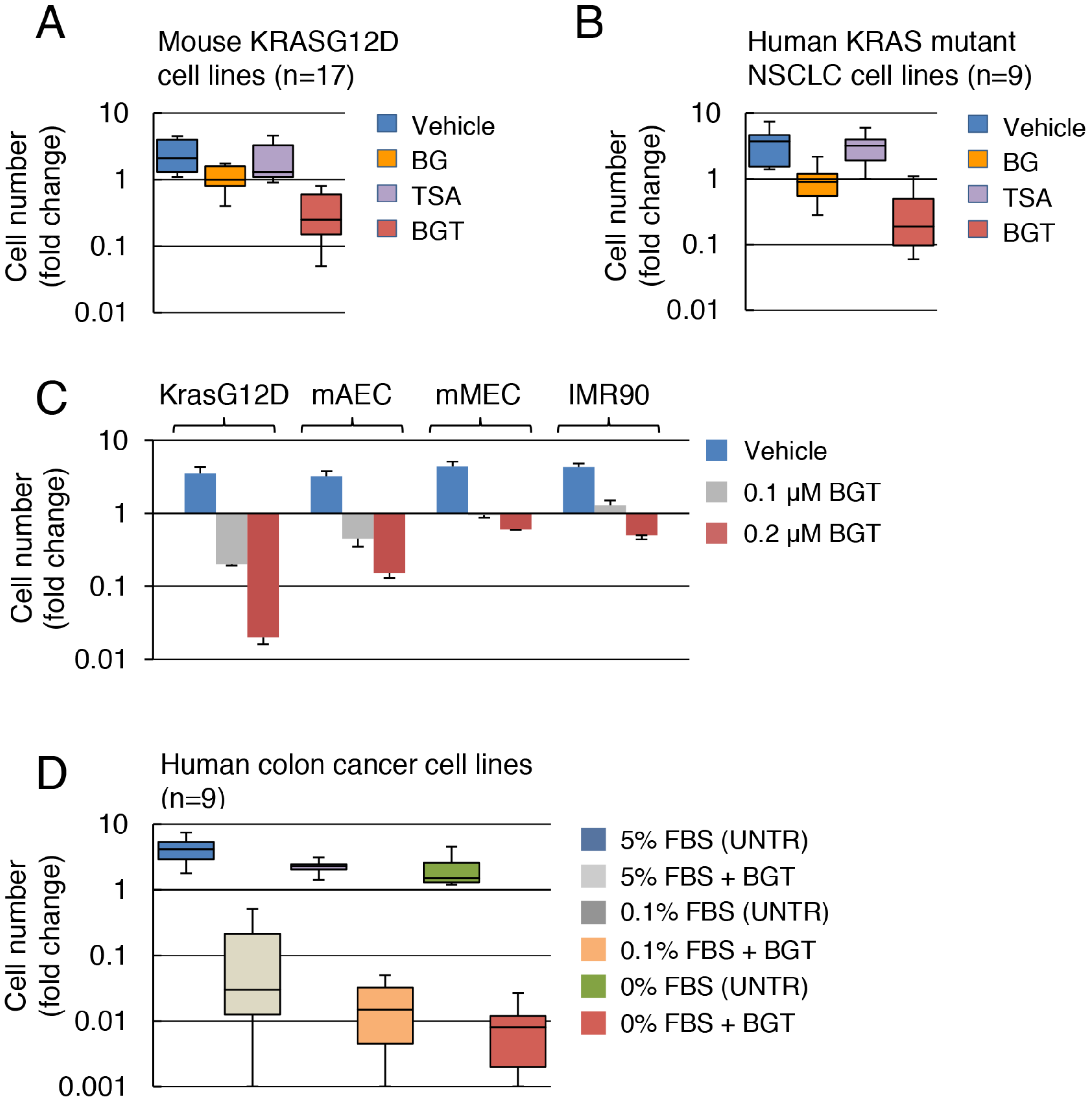

Supplementary Figure 1: Targeting HDACs in combination with KRAS effector pathways overcomes drug resistance in lung cancer cells. A. KRAS G12D cell lines (n=17) were treated with vehicle (DMSO) or the indicated inhibitors at 0.1 µM for 3 d. Fold change in cell numbers relative to input cells is shown. Error bars represent the standard deviation. **B.** Human KRAS mutant NSCLC cell lines (n=8) were treated with vehicle (DMSO) or the indicated inhibitors at 0.1 µM for 3 d. Fold change in cell numbers relative to input cells is shown. Error bars represent the standard deviation. **C.** Normal mouse airway epithelial cells (mAEC), normal mouse mammary epithelial cells (mMEC) and normal human IMR90 lung fibroblasts were treated with BGT inhibitors at 0.1 µM or 0.2 µM for 3 d. Fold change in cell numbers relative to input cells is shown. Mouse KRASG12D airway epithelial cells are shown for comparison. **D.** Colon cancer cell lines (n=9) (C) were maintained in DME medium supplemented with different concentrations of FBS and treated with vehicle or BGT at 0.1 µM for 3 d. Fold change in cell numbers relative to input cells is shown. Error bars represent the standard deviation.

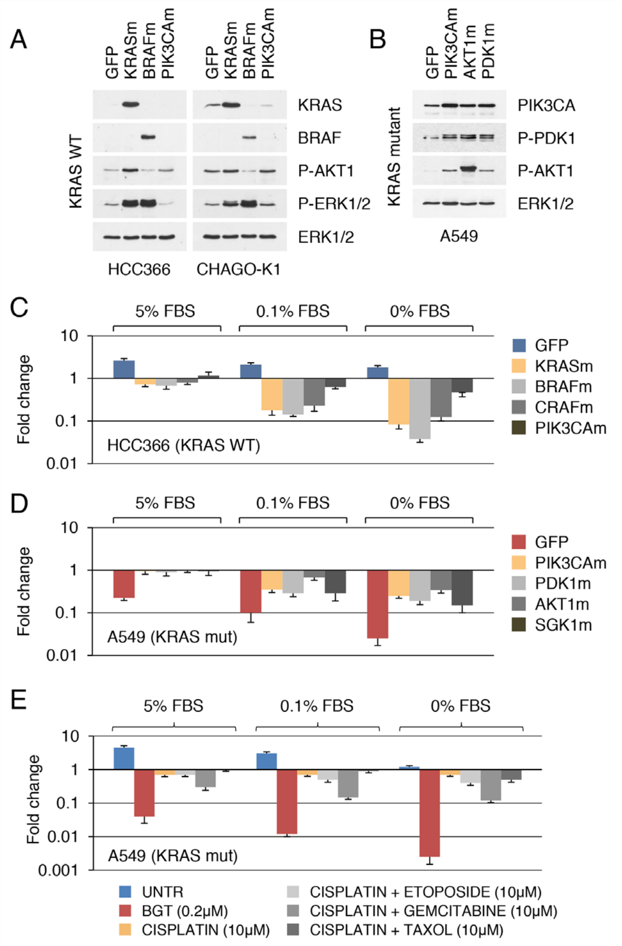

Supplementary Figure 2: Ectopic expression of oncogenic KRAS confers enhanced drug sensitivity on NSCLC cell lines. To assess the contribution of KRAS and its downstream effectors to the drug sensitivity versus resistance, we used NSCLC cell lines stably transduced with constitutively active KRAS G12D, BRAF V600E, CRAF 22W and PIK3CA H1047R mutants (Supplementary Figure 2A, B). The KRAS G12D, BRAF V600E and, to a lesser extent, CRAF 22W mutant conferred drug sensitivity on KRAS/BRAF wild-type NSCLC cells, while having less effect on KRAS mutant cells (Supplementary Figure 2C). In contrast, the activated PIK3CA H1047R or its downstream effectors myr-AKT1, myr-PDK1, and myr-SGK1, conferred an enhanced drug resistance on KRAS mutant NSCLC cells, while having less effect on KRAS WT cells (Supplementary Figure 2D). This analysis further suggests that NSCLC cell lines carrying KRAS mutations are selectively sensitive to MEK and HDAC inhibition. However, KRAS dependency and the ensuing drug sensitivity may be most penetrant in the absence of activating mutations in either upstream (PIK3CA) or downstream (PDK1, AKT1, SGK1) effectors of the PI3K pathway. To gain additional insights into the role of PI3K in drug resistance, we measured the effects of MEK/PI3K/HDAC inhibition on a panel of lung and colorectal carcinoma cells carrying single and compound KRAS, BRAF and PI3K mutations (Supplementary Table 1). We observed a relatively uniform response across all cell lines tested, as dual MEK/PI3K inhibition was cytotoxic in a minority of cells, while maximal induction of cell death occurred with the triple BGT drug combination. Among the cells lines used in our analysis, two (H460 and HCT116) were co-mutated for KRAS and PIK3CA, while two (RKO and HT29) were co-mutated for BRAF and PIK3CA, and two (H23 and CT26) were co-mutated for KRAS and PIK3CG (COSMIC). The triple combination (BGT), but not the dual combination (BG), had a measurable cytotoxic activity against both KRAS/PI3K mutants and BRAF/PI3K mutants, as well as those without dual mutations. Similar to the results described above, we observed a sizable (7-fold) decrease in viability of BGT-treated CRC cells at low serum concentrations. These data imply that the BGT inhibitor combination is reasonably toxic to KRAS mutant cancer cell lines regardless of tumor type. **A, B.** Western blot analysis of total cell lysates from human NSCLC cell lines stably transduced with KRAS G12D (KRASm), BRAF V600E (BRAFm), PIK3CA H1047R (PIK3Cam), myr-AKT1 (AKT1m) and myr-PDK1 (PDK1m). **C.** Human NSCLC cell lines with stable expression of GFP, KRAS G12D, BRAF V600E or CRAF 22W were maintained in DME supplemented with different concentrations of FBS and treated with vehicle alone or BGT inhibitors at 0.1 μM for 3d. Fold change in cell numbers relative to input cells is shown. C. Human HCC366 cells with stable expression of GFP, KRAS G12D, BRAF V600E or CRAF 22W were maintained in DME supplemented with different concentrations of FBS and treated with vehicle alone or BGT inhibitors at 0.1 μM for 3d. Fold change in cell numbers relative to input cells is shown. **D.** Human A549 cells with stable expression of GFP, PIK3CA H1047R, myr-AKT1, myr-PDK1 or myr-SGK1 were maintained in DME supplemented with different concentrations of FBS and treated with vehicle alone or BGT inhibitors at 0.1 μM for 3d. Fold change in cell numbers relative to input cells is shown. **E.** A549 cells were maintained in DME supplemented with different concentrations of FBS and treated for 3 d with BGT at 0.2 μM or cisplatin, cisplatin plus gemcitabine, cisplatin plus taxol or cisplatin plus etoposide at 10 μM each. Fold change in cell numbers relative to input cells is shown.

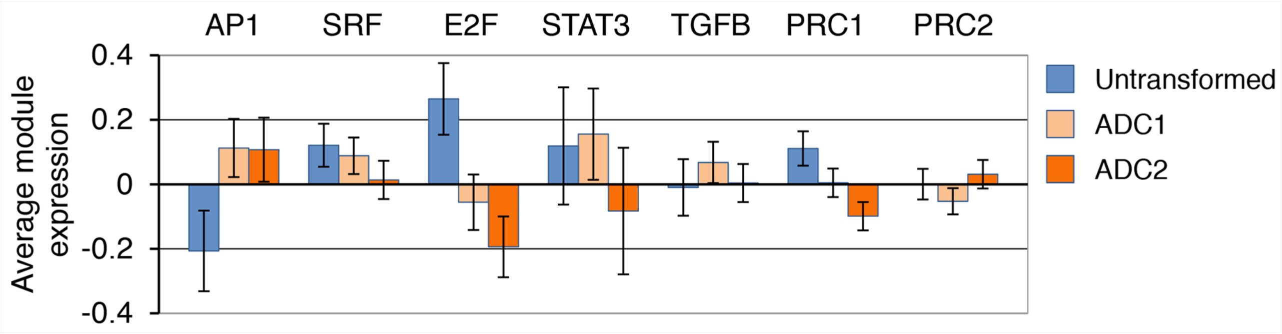

Supplementary Figure 3: Functional separation between resistance mechanisms and KRAS mutation status. Average gene expression values (log2) for each of the indicated modules in normal lung epithelial cells, primary and secondary KRAS G12D lung adenocarcinomas (ADC1 and ADC2). Error bars represent the standard deviation.

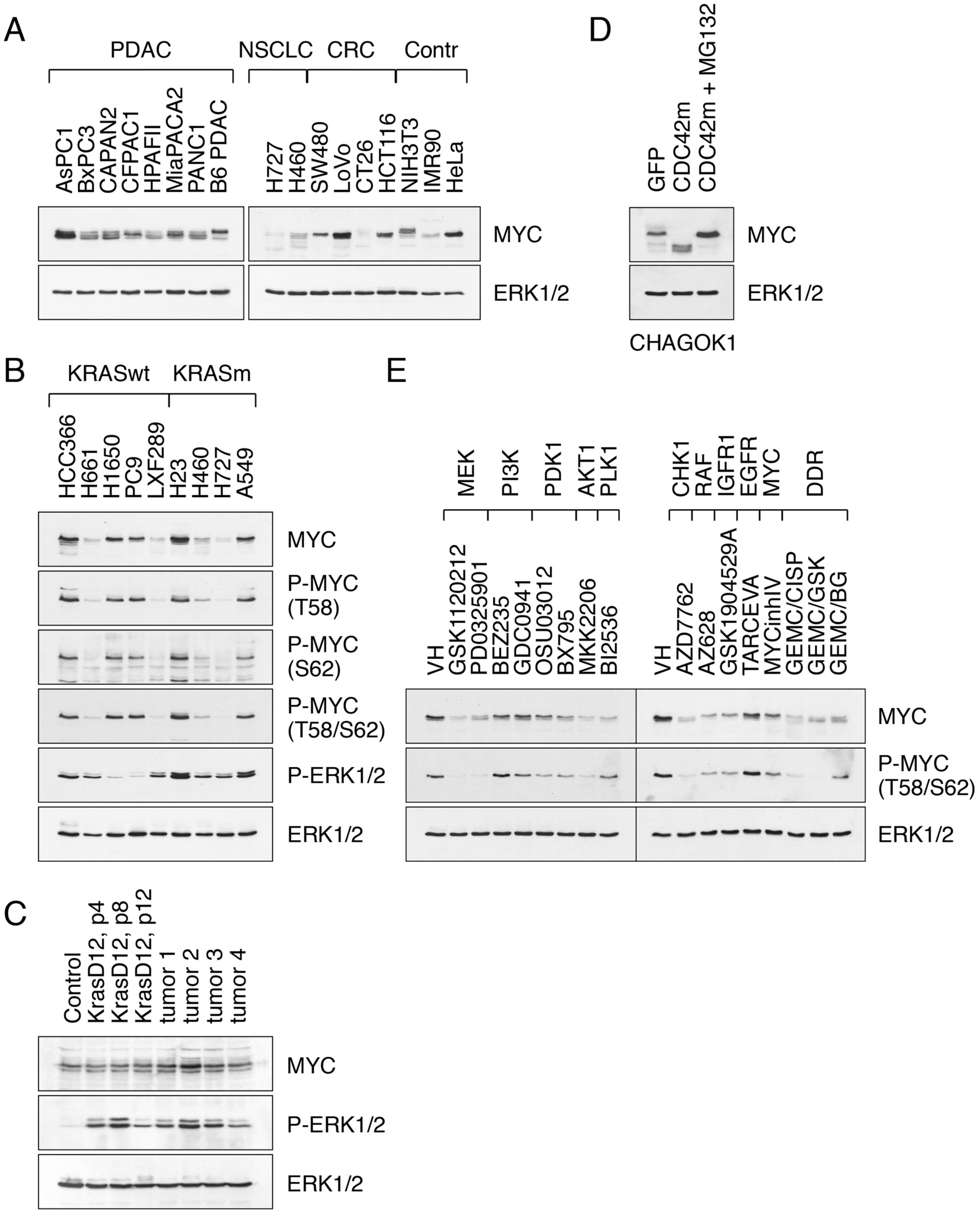

Supplementary Figure 4: Constitutive activation of KRAS does not sustain high levels of MYC. A. MYC expression in nuclear extracts from PDAC and CRC cell lines. Extracts from NIH3T3, IMR90 and HeLa cells, which express different levels of MYC protein (10,000-30,000 molecules per cell, respectively) are shown for comparison. **B**. Western blot analysis of MYC, S62-MYC, T58-MYC and T58/S62-MYC expression in nuclear extracts from KRAS WT and mutant NSCLC cell lines. **C.** MYC expression in whole cell lysates from LSL KRAS G12D p53KO (inactive KRAS G12D allele) and KRAS G12D p53KO (active KRAS G12D allele) lung epithelial cells or tumors derived from these cells. Passage numbers are indicated. **D.** MYC expression in nuclear extracts from CHAGO-K1 cells stably transduced with GFP or CDC42 Q61L (CDC42m). Cells were incubated for 10 h in the presence or absence of the proteasome inhibitor MG132 (5µM). **E.** Western blot analysis of MYC expression in nuclear extracts from A549 cells treated with the indicated inhibitors at 0.1 µM for 3 d. Cellular targets for each inhibitor are indicated. Gemcitabine and Cisplatin (DNA damage response, DDR) were used at 10 µM each.

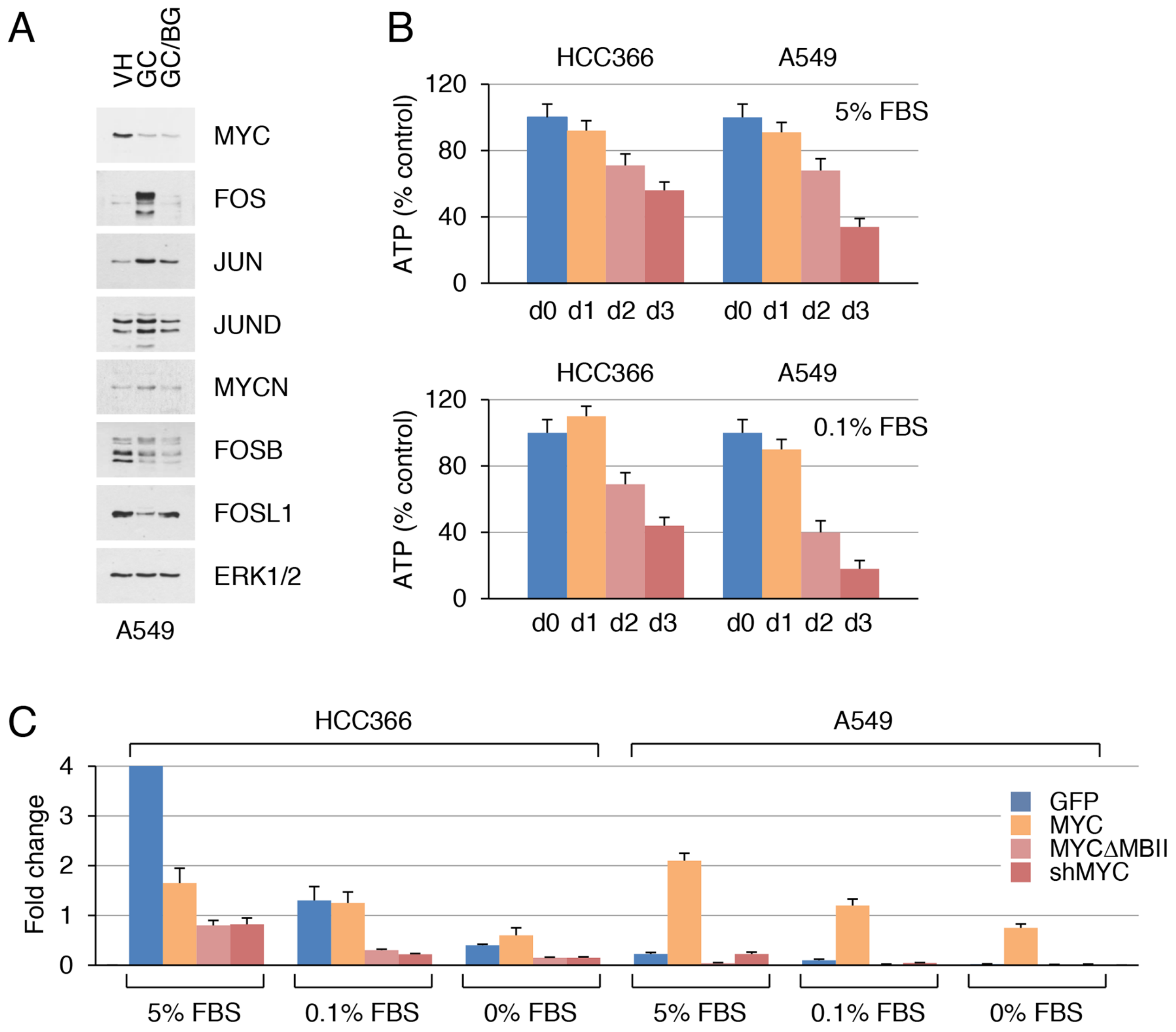

Supplementary Figure 5: Drug-induced cytotoxicity of KRAS mutant cancer cells is contingent on MYC inhibition. A. Western blot analysis of nuclear extracts from A549 cells treated with vehicle alone or GC at 5 µM plus BEZ235/GSK1120212 at 0.1 µM each for 2 d. **B.** Comparison of intracellular ATP contents in HCC366 and A549 cells treated with GC at 5 µM for 3 d. Cells were maintained in DME supplemented with 5% or 0.1% FBS. **C.** Human HCC366 and A549 cells with stable expression of GFP, MYC, MYC ΔMBII or shMYC were maintained in DME supplemented with different concentrations of FBS and treated with vehicle alone or BGT inhibitors at 0.1 µM for 3d. Fold change in cell numbers relative to input cells is shown.

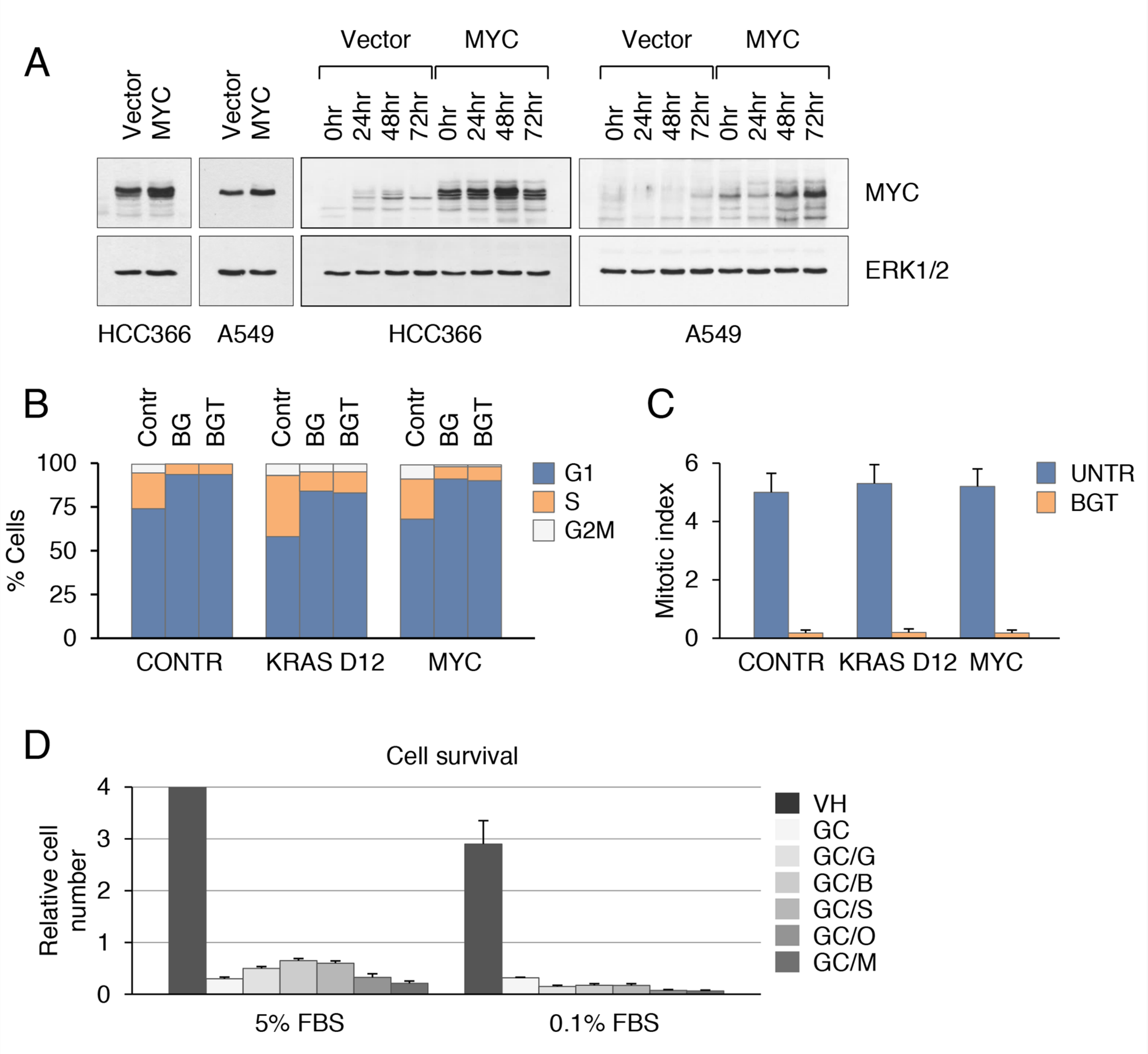

Supplementary Figure 6: Activation of ERK facilitates MYC suppression under drug induced stress conditions. A. Left panels: Western blot analysis of MYC expression in nuclear extracts from vector or MYC-transduced HCC366 and A549 cells. Right panels: Vector or MYC-transduced HCC366 and A549 cells were maintained in DME supplemented with 0.1% FBS and treated with BGT inhibitors at 0.1 µM each for 3 d and then released into drug-free medium (0.1% FBS) for the indicated periods of time. **B.** Cell cycle distribution in KRAS G12D and MYC-transduced HCC366 cells treated with BEZ235/GSK1120212 (BG) or BEZ235/GSK1120212/TSA (BGT) at 100 µM each for 3 d. **C.** For the evaluation of mitotic activity, cells were infected with GFP-tagged histone H2B expressing retroviruses and treated with drugs as in (A). Mitotic indices were evaluated as the number of mitotic figures per 100 cells in 10 fields. Results are representative of four NSCLC cell lines. **D.** A549 cells were maintained in DME supplemented with different concentrations of FBS in the presence or absence of gemcitabine plus cisplatin (GC, 5 µM each) and 100 µM GSK1120212 (GC/G), BEZ235 (GC/BG), SCH772984 (GC/S), OSU03012 (GC/O) or MKK2206 (GC/M) for 3 d; VH, vehicle. Fold change in cell numbers relative to input cells is shown.

Supplementary Table 1: Summary of cancer cell lines used in study.

See Supplementary File 1
