# Supplementary material for: KRAS-dependent suppression of MYC enhances the sensitivity of cancer cells to cytotoxic agents
Source: Oncotarget. 2017 Feb 1;8(11):17995–8009. doi: 10.18632/oncotarget.14929 (PMC5392302; doi:10.18632/oncotarget.14929)
Supplement: Supplementary file 2 [file oncotarget-08-17995-s002.docx]

Supplementary Table 1. Summary of cancer cell lines used in study

***Lung cancer cell lines***

| Cell line | BRAF | CDKN2A | EGFR | KRAS | MET | NRAS | PIK3 | P53 |
| --- | --- | --- | --- | --- | --- | --- | --- | --- |
| H23 | wt | wt | wt | mut | wt | wt | wt | mut |
| H358 | wt | wt | wt | mut | wt | wt | wt | del |
| H460 | wt | wt | wt | mut | wt | wt | mut  PIK3CA | wt |
| H727 | wt | wt | wt | mut | wt | wt | wt | mut |
| A549 | wt | del | wt | mut | wt | wt | wt | wt |
| HOP62 | wt | wt | wt | mut | wt | wt | wt | mut |
|  | wt | wt | wt | mut | wt | wt | wt | mut |
| RERFLC-AD1 | mut | ND | wt | mut | wt | wt | wt | wt |
| H661 | wt | mut | wt | wt | wt | wt | mut  PIK3C3 | mut |
| H1299 | wt | wt | wt | wt | wt | mut | wt | mut |
| PC9 | wt | ND | mut | wt | ND | ND | ND | ND |
| H1650 | wt | wt | mut | wt | wt | wt | mut  PIK3C2 | mut |
| H1703 | wt | wt | wt | wt | wt | wt | mut  PKI3C2 | mut |
| HCC366 | wt | wt | wt | wt | wt | wt | mut  PIK3R1 | mut |
| EBC1 | wt | wt | wt | wt | amplif | wt | mut  PIK3R2 | mut |
| H1838 | wt | wt | wt | wt | mut | wt | wt | mut |
| H2023 | wt | wt | wt | wt | wt | wt | wt | mut |
| H2228 | wt | wt | wt | wt | wt | wt | wt | mut |
| LCLC103H | wt | wt | wt | wt | wt | wt | wt | mut |
| LXF289 | wt | wt | wt | wt | wt | wt | wt | mut |
| CHAGO K1 | wt | wt | wt | wt | wt | wt | wt | mut |
| RERFLC-SQ1 | wt | ND | ND | ND | ND | ND | ND | ND |
| COLO699 | ND | ND | ND | wt | ND | ND | ND | ND |

***Pancreatic cancer cell lines***

| Cell line | BRAF | CDKN2A | KRAS | P53 | SMAD4 |
| --- | --- | --- | --- | --- | --- |
| AsPC1 | wt | del | mut | mut | mut |
| BxPC3 | mut | wt | wt | mut | del |
| CAPAN2 | wt | del | mut | mut | wt |
| CFPAC1 | wt | wt | mut | mut | del |
| HPAFII | wt | mut | mut | mut | wt |
| MiaPACa2 | wt | del | mut | mut | wt |
| PANC1 | wt | del | mut | mut | wt |

***Colon cancer cell lines***

| Cell line | BRAF | CDKN2A | KRAS | PIK3 | P53 |
| --- | --- | --- | --- | --- | --- |
| HCT116 | wt | mut | mut | wt | wt |
| HCT116 (b) | wt | mut | mut | wt | del |
| CT26 | ND | del | mut | ND | wt |
| LoVo | wt | wt | mut | wt | wt |
| SW480 | wt | wt | mut | wt | mut |
| CACO2 | ND | ND | mut | ND | mut |
| RKO | mut | wt | wt | mut | wt |
| HT29 | mut | wt | wt | mut | mut |
| HCA7 | ND | ND | wt | ND | mut |
| COGA10 | ND | ND | wt | ND | wt |

Mutational information on the cancer cell lines was obtained from the COSMIC Library (Catalogue of Somatic Mutations in Cancer) provided by the Sanger Institute (<http://cancer.sanger.ac.uk/cosmic>) and some papers. HCT116(a), TP53 WT; HCT116 (b), TP53-null; ND, not determined.
